# Assessment of the effects of whole-body muscle and fat mass on bone mineral content based on 628 DXA datasets

**DOI:** 10.3389/fendo.2026.1798429

**Published:** 2026-03-11

**Authors:** Jing Liu, Dongpan Chen, Tanli Min, Ling Bai, Xin Zhang, Sheng Wang, Zun Wang, Mai Chen, Biyun Zhang, Daoming Xu

**Affiliations:** 1 Affiliated Hospital of Nanjing University of Chinese Medicine, Jiangsu Province Hospital of Chinese Medicine, Nanjing, China; 2 School of Acupuncture-Moxibustion and Tuina, Nanjing University of Chinese Medicine, Nanjing, China; 3 School of Health Preservation and Rehabilitation, Nanjing University of Chinese Medicine, Nanjing, China; 4 Zhimaying Community Health Center, Qinhuai, Nanjing, China

**Keywords:** body composition, bone mineral content, data analysis, fat mass, muscle mass

## Abstract

**Background:**

The prevalence of osteoporosis is steadily increasing. Although the relationships between muscle mass, fat mass, and bone mineral content (BMC) have been extensively studied, they remain contentious. This study aimed to develop a unified statistical model to evaluate the independent effects of total muscle and fat mass on BMC, clarify their respective roles in osteoporosis pathogenesis, and inform targeted prevention strategies.

**Methods:**

We retrospectively analyzed data from 628 subjects who underwent body composition assessment at the Affiliated Hospital of Nanjing University of Chinese Medicine between January 2022 and July 2024. Collected parameters included age, height, weight, body mass index (BMI), total BMC, total muscle mass, total fat mass, and whole-body bone mineral density (BMD) T-scores. Groups were compared by sex, age and whole-body BMD T-score categories. Spearman correlation and multiple linear regression analyses were used to assess the associations of total BMC with total muscle and total fat mass.

**Results:**

Males had greater height, weight, BMI, total BMC, total muscle mass, and whole-body T-scores than females (*P* < 0.001), while females had higher total fat mass. Total muscle mass consistently showed a stronger positive correlation with total BMC than total fat mass across all subgroups. Multiple linear regression analysis confirmed total muscle mass was the strongest and stable independent positive correlate of total BMC(overall sample *β=*0.687, *P* < 0.001). Total fat mass was positively associated with total BMC only in females, individuals >70 years, and osteoporosis patients, not in normal/osteopenic groups. Age negatively correlated with total BMC, especially in females (*β=*-0.395, *P* < 0.001). BMI correlated with lower total BMC in the total sample, females, and osteopenia group.

**Conclusions:**

Muscle mass is the strongest and most consistent correlate of BMC. The role of fat is context-specific, varying by sex, age, and bone health. Based on current associative evidence, maintaining muscle may be considered a priority in bone health management, while fat’s impact requires individualized assessment.

**Clinical Trial Registration:**

http://www.chictr.org.cn/ identifier ChiCTR2400085209.

## Introduction

Osteoporosis is a systemic metabolic bone disease characterized by reduced bone mineral content and microarchitectural deterioration, leading to increased bone fragility and fracture risk. Fragility fractures are among its most serious complications. Clinical guidelines commonly recommend osteoporosis screening in postmenopausal women and men aged 50 years and older (1). Bone mineral density (BMD) peaks in early adulthood and declines with age, with postmenopausal women experiencing accelerated bone loss (2). In China, osteoporosis affects 6.9% of men and 32.1% of women over 50, and the burden of fragility fractures ranks second highest worldwide (3). Dual-energy X-ray absorptiometry (DXA) remains the gold standard for diagnosing osteoporosis (3), and is increasingly used to evaluate body composition, including muscle and fat mass, in conditions such as sarcopenia and obesity (4, 5).

An intimate functional and metabolic relationship exists between muscle and bone. Mechanical loading from muscle contractions stimulates bone formation through mechanosensitive pathways involving osteocyte signaling (6–8). Muscle mass is a strong predictor of bone mineral content (BMC) (2), and muscle-derived factors such as irisin and insulin-like growth factor-1(IGF-1) promote bone formation, while myostatin inhibits it (9, 10). In addition, muscle-secreted cytokines and extracellular vesicles carrying microRNAs can directly influence bone remodeling (11, 12). Conversely, bone-derived osteocalcin modulates muscle metabolism, highlighting a bidirectional crosstalk (2). These interactions underscore the importance of muscle maintenance in osteoporosis prevention.

Although areal bone mineral density (aBMD) is the standard clinical parameter for diagnosing osteoporosis and assessing fracture risk (1, 3), it may not be the most appropriate measure for investigating the relationship between body composition and bone. aBMD is mathematically derived by dividing bone mineral content (BMC) by bone area, rendering it inherently influenced by skeletal size (13, 14). Given that body size correlates strongly with both muscle and fat mass, using aBMD as the outcome could introduce a degree of circularity when evaluating muscle–bone or fat–bone associations. In contrast, BMC represents the absolute mineral content of the skeleton and is less confounded by bone dimensions, thereby providing a more direct reflection of the total mineralized response to mechanical and metabolic stimuli (2, 6). Therefore, to better delineate the independent contributions of muscle and fat mass to skeletal mineral status, this study employed total BMC as the primary outcome measure.

In contrast, the role of fat mass in bone metabolism remains controversial. While some studies suggest a protective mechanical effect of adipose tissue on bone density (15–17), others report that excess fat-particularly visceral fat-promotes inflammation and secretion of adipokines such as leptin and tumor necrosis factor-α(TNF-α), which may suppress bone formation and enhance resorption (18). Most prior studies have assessed muscle or fat in isolation, limiting the ability to discern their independent effects on bone.

To address this gap, we analyzed body composition data from 628 clinical subjects to evaluate the independent contributions of total muscle mass and total fat mass to BMC within a unified statistical model. Furthermore, we stratified analyses by sex and bone density status to clarify population-specific effects. This approach provides a more integrated perspective on muscle-fat-bone interactions and supports the development of tailored strategies for osteoporosis prevention and management.

## Materials and methods

### Study population and design

**Figure 1:**
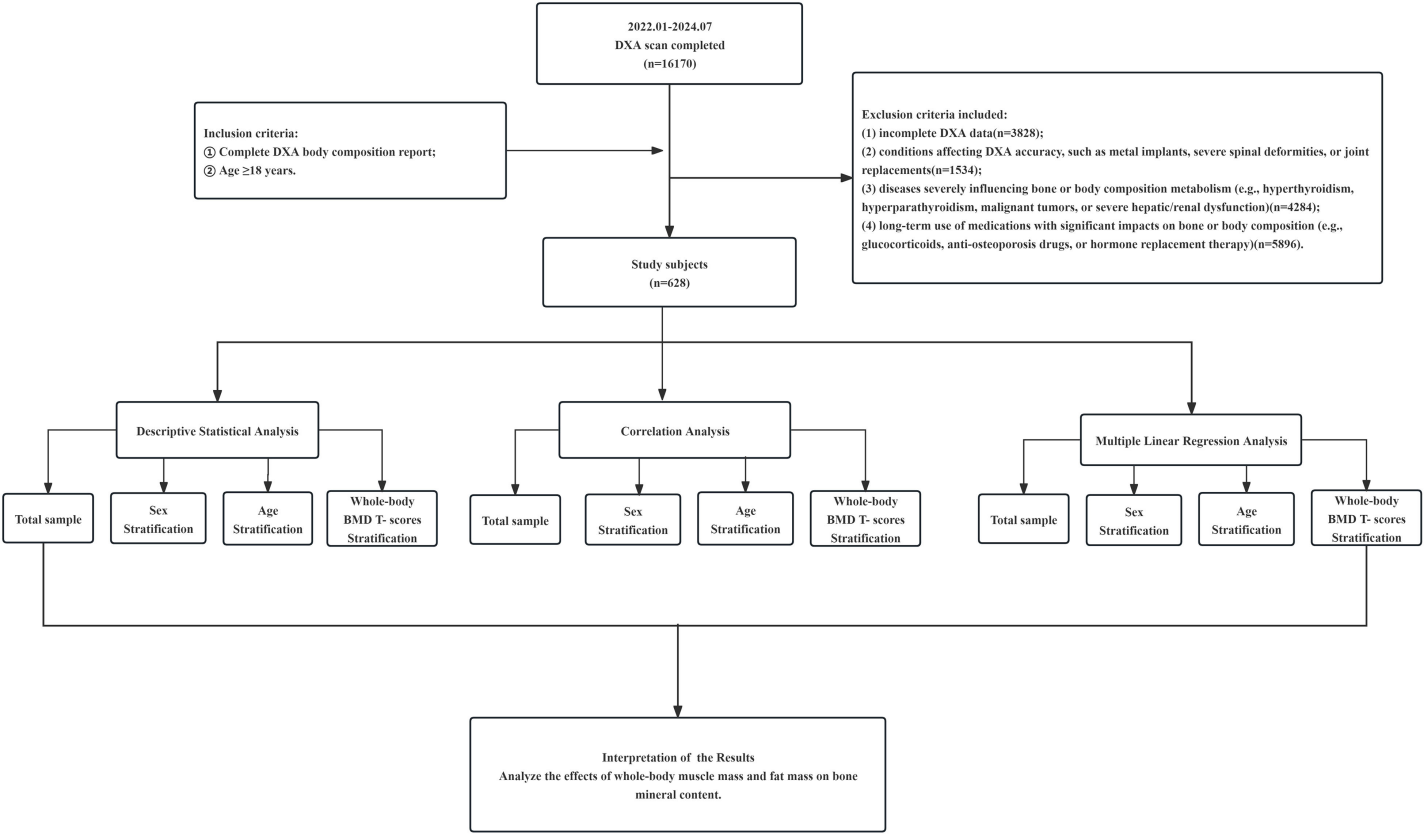
Flowchart of the study.

This retrospective study screened individuals who underwent DXA body composition examination at the Department of Nuclear Medicine, Affiliated Hospital of Nanjing University of Chinese Medicine, between January 2022 and July 2024.The detailed procedures of this study are described in the Flowchart (**Figure 1**). The participants were primarily outpatients and inpatients who voluntarily underwent DXA examination for health assessment or clinical indication.

Inclusion criteria were: (1) Availability of a complete DXA report; (2) Age ≥ 18 years.

Exclusion criteria included: (1) Incomplete DXA data(*n=*6,321 excluded); (2) Conditions affecting DXA accuracy, such as metal implants, severe spinal deformities, or joint replacements (*n=*1, 582 excluded); (3) Diseases severely influencing bone or body composition metabolism (e.g., hyperthyroidism, hyperparathyroidism, Cushing’s syndrome, malignant tumors, or severe hepatic/renal dysfunction)(*n=*7,125 excluded); (4) Long-term use of medications with significant impacts on bone or body composition (e.g., glucocorticoids, anti-osteoporosis drugs, or hormone replacement therapy) (*n* = 514 excluded).

A total of 16,170 individuals were initially identified. After applying the eligibility criteria, 628 subjects were included in the final analysis. The study was approved by the Ethics Committee of the Affiliated Hospital of Nanjing University of Chinese Medicine (No. 2024NL-085-02, May 9, 2024) and registered with the Chinese Clinical Trial Registry (ChiCTR2400085209||http://www.chictr.org.cn/, June 3, 2024).

### Data collection and measurements

Demographic and body composition data were retrieved from the Clinical Information System of the Department of Nuclear Medicine. Collected variables included age, height, body weight, body mass index (BMI), total BMC, total muscle mass (representing DXA-derived lean soft tissue mass), total fat mass, and whole-body BMD T-score. BMI was calculated as weight in kilograms divided by the square of height in meters (kg/m²). All measurements were performed using a Hologic Discovery DXA bone densitometer (Model ASY-03954). It should be noted that the “total muscle mass” measured by DXA technically represents lean soft tissue mass, which includes water, connective tissue, and organ mass in addition to skeletal muscle. Similarly, “total fat mass” represents whole-body adipose tissue without distinguishing between subcutaneous and visceral depots. All measurements reported herein are whole-body values. In this analysis, BMC was selected as the primary outcome because it reflects the total amount of mineral in the skeleton and is less influenced by bone size compared to a real BMD, thereby providing a more direct measure of bone mass in relation to body composition. This choice also minimizes the risk of partial circularity when interpreting associations with muscle mass, as aBMD is mathematically derived using bone area which correlates with body size. Given that sex and age are established determinants of BMC, and skeletal health status represents a complete spectrum from normality to disease, this study conducted subgroup analyses to comprehensively evaluate the characteristics of the associations between muscle mass, fat mass, and BMC across different populations and health states.

### Statistical analysis

Data were analyzed using SPSS Statistics (Version 29.0). Normally distributed continuous variables are presented as mean ± standard deviation, and non-normally distributed variables as median and interquartile range. Group comparisons were conducted using ANOVA for normally distributed data(after confirmation by Shapiro-Wilk test), and the Mann-Whitney U or Kruskal-Wallis test for non-normal data. Correlations between variables were assessed using Spearman’s rank correlation analysis, and correlation heatmaps were generated with GraphPad Prism (Version 10). To assess the robustness of the primary associations and to perform a sensitivity analysis, two multiple linear regression models were constructed. Model 1 included only total muscle mass and total fat mass to examine their unadjusted associations with total BMC. Model 2 further adjusted for age and BMI as covariates. The stability of the regression coefficients for muscle mass and fat mass across the two models was evaluated to determine the sensitivity of these core associations to model specification. Multicollinearity was assessed using variance inflation factor (VIF), with values <5 considered acceptable (all VIFs in the final models were <4.5). A two-sided *P*-value <0.05 was considered statistically significant.

## Results

### Basic characteristics and group comparisons of the included population

A total of 628 participants were enrolled in this study. The collected data were stratified by sex, age, and total body BMD T-score for comparative analysis. Sex-based comparison revealed that males had significantly greater height, weight, BMI, total BMC, total muscle mass, and T-score than females (*P* < 0.001). Conversely, total fat mass was significantly higher in females (*P* < 0.001). No significant difference in age was observed between the two groups (*P* = 0.953) (**Table 1**).

**Table 1:** Baseline characteristics of the included study population and comparisons stratified by sex [M(P25, P75)].

Variable	Total (*n* = 628)	Female (*n* = 498)	Male (*n* = 130)	Z	*P*
Age (year)	61.00 (54.00,69.00)	61.00 (54.00,69.00)	61.50 (53.00,70.00)	-0.059	0.953
Height (cm)	160.00 (156.00,165.00)	159.00 (155.00,163.00)	170.00 (166.75,175.00)	-14.801	<0.001
Weight (kg)	60.00 (54.00,69.00)	59.25 (53.00,65.00)	70.00 (64.80,76.00)	-10.290	<0.001
BMI	23.50 (21.37,25.71)	23.24 (21.25,25.63)	24.23 (22.28,25.82)	-2.480	0.013
Total BMC (g)	1904.48 (1666.51,2259.27)	1819.75 (1591.66,2056.99)	2611.27 (2298.78,2881.55)	-14.590	<0.001
Total Muscle mass (g)	34209.50 (30456.05,39576.78)	32606.40 (29778.58,36160.95)	45298.25 (41150.30,49388.45)	-3.928	<0.001
Total Fat mass (g)	23185.00 (19946.00,27378.25)	23646.50 (20115.50,27768.25)	21723.50 (17101.25, 25143.00)	-15.513	<0.001
T-Score	-0.60 (-1.80,0.50)	-0.90 (-2.10,0.20)	0.25 (-0.63,1.40)	-7.494	<0.001

Age-stratified comparison showed significant differences among the three age groups (<60 years, 60–70 years, >70 years) in age, height, total BMC, and T-score (*P* < 0.001). *Post-hoc* analysis indicated a declining trend in height, total BMC, and T-score with advancing age. Both the 60–70 years and >70 years groups showed significantly lower values compared to the <60 years group. In contrast, no significant differences were found among the groups in weight, BMI, total fat mass, or total muscle mass (*P*>0.05) (**Table 2**).

**Table 2:** Age-stratified comparison [M(P25, P75)].

Variable	<60 years (*n=*271)	60–70 years (*n=*229)	>70 years (*n=*128)	H	*P*
Age (year)	53.00 (48.00,56.00)	65.00^**^ (62.00,68.00)	74.00^**##^ (72.00,77.75)	541.422	<0.001
Height (cm)	161.00 (158.00,167.00)	160.00^*^ (155.00,165.00)	159.00^##^ (155.00,165.00)	14.334	<0.001
Weight (kg)	61.00 (55.00,70.00)	60.00 (54.00,67.50)	60.00 (53.00,70.00)	2.125	0.346
BMI	23.31 (21.23,25.60)	23.53 (21.48,25.90)	23.71 (21.52,25.71)	1.397	0.497
Total BMC (g)	2054.07 (1839.50,2379.43)	1819.07^**^ (1587.14,2111.58)	1783.99^##^ (1495.63,2199.09)	54.629	<0.001
Total Muscle mass (g)	34246.10 (30548.30,40487.90)	34206.40 (30464.30,37911.65)	34049.60 (30167.78,39579.48)	0.561	0.756
Total Fat mass (g)	23485.00 (20137.00,27381.00)	22623.00 (19522.50,27071.00)	23084.00 (19058.75,27900.00)	3.396	0.183
T-Score	-0.20 (-1.00,0.80)	-1.10^**^ (-2.20,0.00)	-1.40^**^ (-2.50,0.18)	57.948	<0.001

Compared with the <60 years group *P < 0.05, **P < 0.01.

Compared with the 60–70 years group: ## for *P* < 0.01.

Comparison based on whole-body BMD T-score demonstrated that, compared to the normal bone mass group, both the osteopenia and osteoporosis groups were characterized by significantly increased age and decreased height, total BMC, total muscle mass, and T-score. Furthermore, compared to the osteopenia group, the osteoporosis group exhibited significantly greater age and lower height, weight, BMI, total BMC, total muscle mass, and T-score (**Table 3**).

**Table 3:** Group comparisons based on whole-body BMD T-score [M(P25, P75)].

Variable	Normal bone mass (*n=*372)	Osteopenia (*n=*163)	Osteoporosis (*n=*93)	H	*P*
Age (year)	58.00 (52.00,66.00)	65.00^***^ (57.00,70.00)	67.00^***#^ (62.00,74.00)	66.554	<0.001
Height (cm)	162.00 (158.00,168.00)	160.00^***^ (155.00,164.00)	157.00^***##^ (153.00,160.00)	62.444	<0.001
Weight (kg)	62.25 (55.00,70.00)	60.00 (55.00,67.00)	55.00^***###^ (51.00,60.00)	44.621	<0.001
BMI	23.66 (21.48,25.77)	23.78 (21.51,26.06)	22.31^**##^ (20.76,24.45)	13.686	0.001
Total BMC (g)	2177.51 (1926.27,2522.26)	1717.33^***^ (1604.33,1830.06)	1416.63^***###^ (1289.03,1532.23)	378.332	<0.001
Total Muscle mass (g)	35854.05 (31218.55,41984.33)	34061.10^**^ (30912.20,37637.80)	30735.80^***###^ (27752.40,34019.65)	55.502	<0.001
Total Fat mass (g)	23247.50 (20078.75,27627.75)	23798.00 (20137.00,27814.00)	22250.00^*##^ (17564.50,25137.50)	9.112	0.011
T-Score	0.20 (-0.40,1.10)	-1.70^***^ (-2.10,-1.30)	-3.10^***###^ (-3.45,-2.70)	478.394	<0.001

Compared with the normal bone mass group^*^P<0.05, ^**^P<0.01, ^***^P<0.001.

Compared with the osteopenia group^#^*P<*0.05, ^##^*P<*0.01, ^###^*P<*0.001.

### Correlation analysis

**Figure 2:**
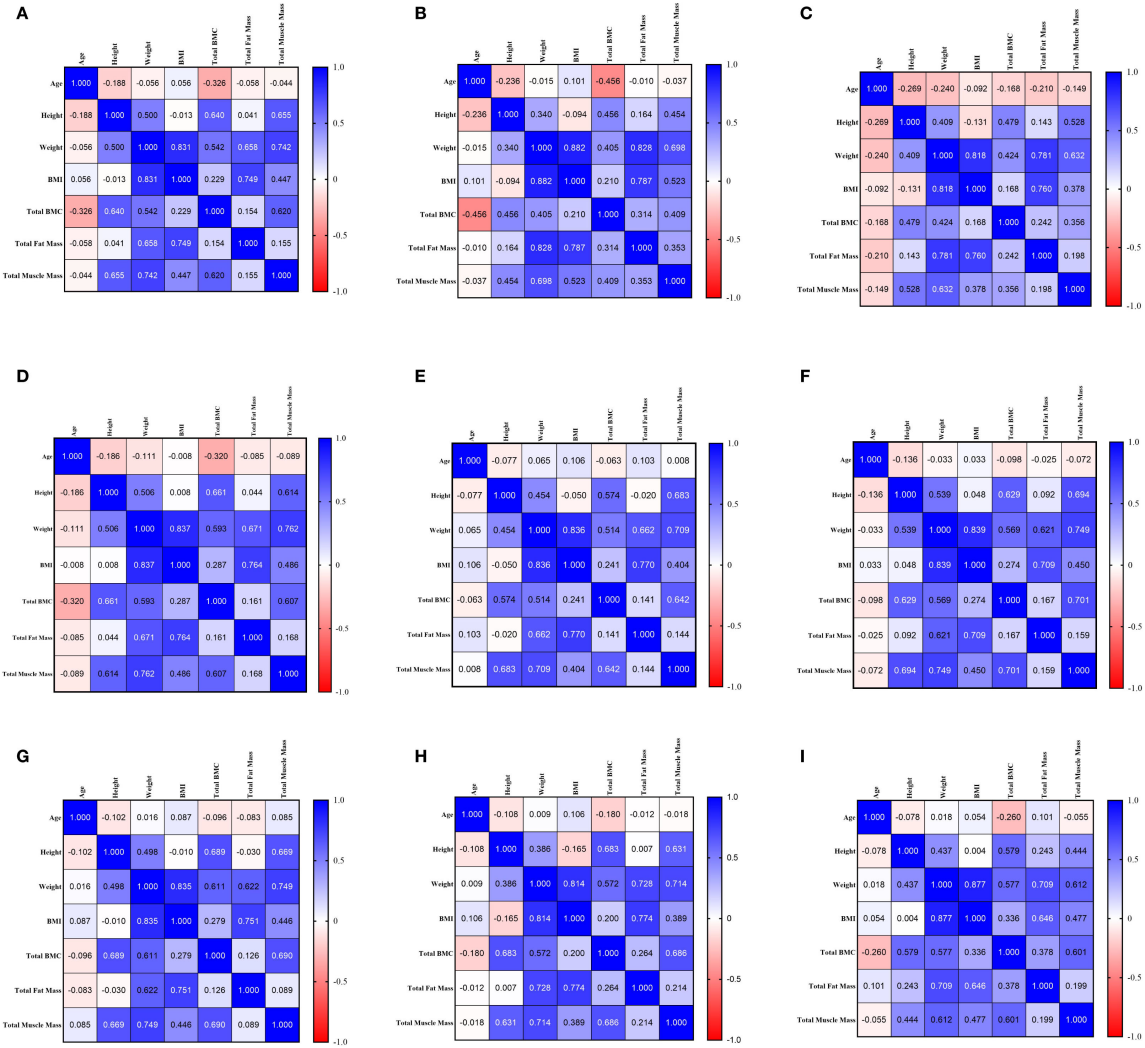
**(a)** Correlation for Total Sample (*n=*628). **(b)** Correlation for Females (*n=*498). **(c)** Correlation for Males (*n=*130). **(d)** Correlation for <60 years (*n=*271). **(e)** Correlation for 60–70 years (*n=*229). **(f)** Correlation for >70 years (*n=*128). **(g)** Correlation for Normal Bone Mass (*n=*372). **(h)** Correlation for Osteopenia (*n=*163). **(i)** Correlation for Osteoporosis (*n=*93).

Spearman correlation analysis was conducted to evaluate the relationships between total BMC and variables including age, height, weight, BMI, total muscle mass, and total fat mass. In the overall sample (*n=*628, **Figure 2a**), Total BMC showed a significant negative correlation with age (*r=*-0.326, *P* < 0.001) and significant positive correlations with height, weight, BMI, total muscle mass, and total fat mass(all *P* < 0.001). The strongest positive correlation was observed with total muscle mass (*r=*0.620), which was substantially higher than that with total fat mass (*r=*0.154).

After stratification by sex, the correlation pattern in females (*n=*498, **Figure 2b**) remained consistent with the total sample, though the inverse correlation between total BMC and age was more pronounced (*r=*-0.456, *P* < 0.001). In males (*n=*130, **Figure 2c**), total BMC was not significantly correlated with age or BMI (*P*>0.05) but showed positive correlations with height, weight, total muscle mass, and total fat mass (all *P* < 0.01). The correlation with total muscle mass in males (*r=*0.356) was weaker than that in females and the overall sample.

Age-stratified analysis demonstrated evolving correlation patterns. In the <60 years group (*n=*271, **Figure 2d**), total BMC was negatively correlated with age (*r=*-0.320, *P* < 0.001) and strongly positively correlated with height (*r=*0.661) and total muscle mass (*r=*0.607) (both *P* < 0.001). The correlation with total fat mass was weak but significant (*r=*0.161, *P* = 0.008). In the 60–70 years group (*n=*229, **Figure 2e**), the correlation between total BMC and age was no longer significant (*r=*-0.063, *P* = 0.343). Strong positive correlations persisted with height (*r=*0.574) and total muscle mass (*r=*0.642) (both *P* < 0.001), while the association with total fat mass was marginal (*r=*0.141, *P* = 0.033). In the >70 years group (*n=*128, **Figure 2f**), total BMC was not significantly associated with age (*r=*-0.098, *P* = 0.273) but remained strongly positively correlated with height (*r=*0.629) and total muscle mass (*r=*0.701) (both *P* < 0.001). The correlation with total fat mass was not significant (*r=*0.167, *P* = 0.059).

When stratified by whole-body BMD T-score, total BMC in the normal bone mass group (*n=*372, **Figure 2g**) showed no significant correlation with age but a strong positive correlation with total muscle mass (*r=*0.690, *P* < 0.001). In both the osteopenia (*n=*163, **Figure 2h**) and osteoporosis (*n=*93, **Figure 2i**) groups, total BMC was negatively correlated with age (*r=*-0.180, *P* = 0.022; *r=*-0.260, *P* = 0.012, respectively) and remained strongly correlated with total muscle mass. Notably, the correlation between total BMC and total fat mass was highest in the osteoporosis group (*r=*0.378).

### Multiple linear regression analysis

### Multiple linear regression analysis of the total sample data

Analysis of the total sample (*n=*628, **Table 4**) indicated that both muscle mass and fat mass were significant predictors of total BMC. In Model 1, total muscle mass (*β=*0.652, *P* < 0.001) was a strong positive predictor, with each 1-gram increase associated with an average increase of 0.044 g in total BMC. Total fat mass (*β=*0.061, *P* = 0.045) also reached statistical significance, although its effect was modest. This model explained 43.8% of the variance in total BMC (adjusted R²=0.438).

**Table 4:** Multiple linear regression analysis of total sample and sex-stratified data.

Group	Model	Variable	B	*β*	t	*P*	95.0% CI for B		Adjusted R^2^
							Lower bound	Upper bound	
Total Sample (*n=*628)	Model 1	(Intercept)	326.126		3.818	<0.001	158.404	493.848	0.438
		Total Fat mass	0.005	0.061	2.012	0.045	0.000	0.009	
		Total Muscle mass	0.044	0.652	21.562	<0.001	0.040	0.048	
	Model 2	(Intercept)	1188.499		9.380	<0.001	939.674	1437.324	0.500
		Total Fat mass	0.012	0.165	3.436	<0.001	0.005	0.020	
		Total Muscle mass	0.046	0.687	20.695	<0.001	0.042	0.051	
		Age	-9.340	-0.218	-7.499	<0.001	-11.785	-6.894	
		BMI	-23.776	-0.169	-3.242	0.001	-38.177	-9.374	
Females (*n=*498)	Model 1	(Intercept)	758.239		8.244	<0.001	577.524	938.953	0.230
		Total Fat mass	0.013	0.227	5.329	<0.001	0.008	0.017	
		Total Muscle mass	0.024	0.347	8.134	<0.001	0.018	0.029	
	Model 2	(Intercept)	1716.763		14.745	<0.001	1487.995	1945.531	0.400
		Total Fat mass	0.017	0.308	5.258	<0.001	0.011	0.023	
		Total Muscle mass	0.024	0.356	8.493	<0.001	0.019	0.030	
		Age	-12.730	-0.395	-11.111	<0.001	-14.981	-10.479	
		BMI	-13.091	-0.133	-2.050	0.041	-25.639	-0.544	
Males (*n=*130)	Model 1	(Intercept)	1354.237		4.471	<0.001	754.880	1953.593	0.126
		Total Fat mass	0.015	0.219	2.602	0.010	0.004	0.027	
		Total Muscle mass	0.021	0.259	3.079	0.003	0.007	0.034	
	Model 2	(Intercept)	1781.819		4.174	<0.001	936.916	2626.722	0.131
		Total Fat mass	0.027	0.383	2.637	0.009	0.007	0.047	
		Total Muscle mass	0.025	0.315	3.395	<0.001	0.010	0.039	
		Age	-0.840	-0.024	-0.275	0.783	-6.879	5.198	
		BMI	-34.041	-0.229	-1.522	0.130	-78.301	10.219	

Dependent variable: Bone mineral content.

Model 1 Predictors: (Constant), Total muscle mass, Total fat mass.

Model 2 Predictors: (Constant), Total muscle mass, Total fat mass, Age, BMI.

B, unstandardized regression coefficient; β, standardized regression coefficient; CI, confidence interval; BMI, body mass index.

After incorporating age and BMI into Model 2, the model’s explanatory power increased to 50.0% (adjusted R²=0.500). The positive predictive effects of total muscle mass (*β=*0.687, *P* < 0.001) and total fat mass (*β=*0.165, *P* < 0.001) remained significant and were notably enhanced. In contrast, age (*β=*-0.218, *P* < 0.001) and BMI (*β=*-0.169, *P* = 0.001) emerged as significant negative predictors. This indicates that each one-year increase in age was associated with an average reduction of 9.340 g in total BMC, and each one-unit increase in BMI was associated with an average reduction of 23.776 g. No multicollinearity issues were detected in either model. The sensitivity analysis confirmed the robustness of these core associations. The positive relationship between muscle mass and BMC remained strong and stable (β increased from 0.652 to 0.687), while the association for fat mass was not only sustained but strengthened after covariate adjustment (β increased from 0.061 to 0.165), as evidenced by the comparison between Model 1 and Model 2.

### Sex-stratified multiple linear regression analysis

Sex-stratified multiple linear regression analysis revealed distinct patterns between sexes. In the female sample (*n=*498, **Table 4**), both total muscle mass (*β=*0.347) and total fat mass (*β=*0.227) in Model 1 exerted significant positive effects on total BMC (*P* < 0.001). Specifically, each 1-gram increase in muscle mass and fat mass was associated with an average increase of 0.024 g and 0.013 g in total BMC, respectively. The model explained 23.0% of the variance in total BMC. After adding age and BMI in Model 2, the explanatory power increased to 40.0%. The effects of total muscle mass (*β=*0.356) and total fat mass (*β=*0.308) remained highly significant (*P* < 0.001). Age (*β=*-0.395, *P* < 0.001) and BMI (*β=*-0.133, *P* = 0.041) were also significant negative predictors. For females, each one-year increase in age was associated with an average reduction of 12.730 g in total BMC, and each one-unit increase in BMI was associated with an average reduction of 13.091 g. In contrast, the male sample (*n=*130, **Table 4**) showed different results. In Model 1, although both total muscle mass (*β=*0.259, *P* = 0.003) and total fat mass (*β=*0.219, *P* = 0.010) were significant predictors, the overall explanatory power of the model was weak (adjusted R²=0.126). The inclusion of age and BMI in Model 2 did not substantially improve the model fit (adjusted R²=0.131). Notably, neither age nor BMI showed a statistically significant effect on total BMC in males (*P*>0.05). Furthermore, the standardized coefficient for total fat mass (*β=*0.383) exceeded that for total muscle mass (*β=*0.315). However, due to the small sample size and low explanatory power of the models in males, these findings should be interpreted with caution.

### Age-stratified multiple linear regression analysis

The age-stratified multiple linear regression analysis yielded the following results. In the <60 years group (*n=*271, **Table 5**), total muscle mass was the sole significant positive predictor of total BMC (Model 1, *β=*0.589; Model 2, *β=*0.604; both *P* < 0.001). After adding age in Model 2, it showed a significant negative effect (*β=*-0.214, *P* < 0.001), with each one-year increase in age associated with an average reduction of 11.716 g in total BMC. The effects of total fat mass and BMI were not significant (**Table 5**). In the 60–70 years group (*n=*229, **Table 5**), total muscle mass exhibited the strongest predictive effect (Model 1, *β=*0.719; Model 2, *β=*0.763; both *P* < 0.001). Within this age range, total fat mass, age, and BMI showed no significant influence on total BMC (**Table 5**). In the >70 years group (*n=*128, **Table 5**), total muscle mass maintained a very strong positive predictive role (Model 1, *β=*0.736; Model 2, *β=*0.792). Notably, after adjusting for age and BMI in Model 2, total fat mass emerged as a significant positive predictor (*β=*0.190, *P* = 0.025), while BMI became a significant negative predictor (*β=*-0.197, *P* = 0.030). The effect of age was not significant (**Table 5**).

**Table 5:** Multiple linear regression analysis stratified by age.

Group	Model	Variable	B	*β*	t	*P*	95.0% CI for B		Adjusted R^2^
							Lower bound	Upper bound	
<60 years (*n=*271)	Model 1	(Intercept)	810.466		6.702	<0.001	572.380	1048.552	0.360
		Total Fat mass	0.005	0.070	1.423	0.156	-0.002	0.011	
		Total Muscle mass	0.034	0.589	11.977	<0.001	0.028	0.039	
	Model 2	(Intercept)	1614.917		8.014	<0.001	1218.148	2011.686	0.408
		Total Fat mass	0.009	0.133	1.493	0.137	-0.003	0.020	
		Total Muscle mass	0.035	0.604	10.253	<0.001	0.028	0.041	
		Age	-11.716	-0.214	-4.366	<0.001	-16.999	-6.433	
		BMI	-14.468	-0.120	-1.230	0.220	-37.635	8.699	
60–70 years (*n=*229)	Model 1	(Intercept)	115.667		0.856	0.393	-150.742	382.076	0.516
		Total Fat mass	0.001	0.013	0.289	0.773	-0.006	0.008	
		Total Muscle mass	0.050	0.719	15.484	<0.001	0.044	0.057	
	Model 2	(Intercept)	707.069		1.463	0.145	-245.115	1659.253	0.520
		Total Fat mass	0.010	0.131	1.670	0.096	-0.002	0.022	
		Total Muscle mass	0.053	0.763	14.587	<0.001	0.046	0.061	
		Age	-6.047	-0.039	-0.845	0.399	-20.149	8.055	
		BMI	-21.978	-0.151	-1.785	0.076	-46.241	2.285	
>70 years (*n=*128)	Model 1	(Intercept)	-266.411		-1.415	0.159	-638.973	106.152	0.549
		Total Fat mass	0.005	0.062	1.036	0.302	-0.004	0.014	
		Total Muscle mass	0.058	0.736	12.257	<0.001	0.048	0.067	
	Model 2	(Intercept)	420.871		0.722	0.472	-733.775	1575.516	0.561
		Total Fat mass	0.015	0.190	2.268	0.025	0.002	0.028	
		Total Muscle mass	0.062	0.792	11.997	<0.001	0.052	0.072	
		Age	-4.459	-0.039	-0.649	0.518	-18.065	9.147	
		BMI	-30.732	-0.197	-2.193	0.03	-58.47	-2.994	

Dependent variable: Bone mineral content.

Model 1 Predictors: (Constant), Total muscle mass, Total fat mass.

Model 2 Predictors: (Constant), Total muscle mass, Total fat mass, Age, BMI.

B, unstandardized regression coefficient; β, standardized regression coefficient; CI, confidence interval; BMI, body mass index.

### Multiple linear regression analysis after stratification by whole-body BMD T-Score

Multiple linear regression analysis based on total body BMD T-score yielded the following group-specific results. In the normal bone mass group (*n=*372, **Table 6**), total muscle mass was the only significant positive predictor in Model 1. After BMI was included in Model 2, it emerged as a significant negative predictor (*β=*-0.245, *P* = 0.001), while the effect of total fat mass also became significantly positive (*β=*0.240, *P* < 0.001). The influence of age was of borderline significance (P = 0.056) (**Table 6**). In the osteopenia group (*n=*163, **Table 6**), total muscle mass showed a stable and strong predictive effect. In Model 2, which included age and BMI, total fat mass (*β=*0.432), age (*β=*-0.142), and BMI (*β=*-0.478) all became highly significant predictors (all *P* < 0.01). The model demonstrated high explanatory power, accounting for 66.0% of the variance in total BMC (**Table 6**). In the osteoporosis group (*n=*93, **Table 6**), both total muscle mass and total fat mass were significant positive predictors in both Model 1 and Model 2. In Model 2, age was a significant negative predictor (*β=*-0.240, *P* = 0.005), whereas the effect of BMI was not significant (**Table 6**).

**Table 6:** Multiple linear regression analysis stratified by whole-body BMD T-score.

Group	Model	Variable	B	*β*	t	*P*	95.0% CI for B		Adjusted R^2^
							Lower bound	Upper bound	
Normal bone mass (*n=*372)	Model 1	(Intercept)	744.630		7.655	<0.001	553.347	935.913	0.460
		Total Fat mass	0.004	0.066	1.726	0.085	-0.001	0.009	
		Total Muscle mass	0.038	0.672	17.549	<0.001	0.034	0.043	
	Model 2	(Intercept)	1147.395		8.386	<0.001	878.333	1416.458	0.484
		Total Fat mass	0.016	0.240	3.537	<0.001	0.007	0.024	
		Total Muscle mass	0.043	0.762	16.763	<0.001	0.038	0.048	
		Age	-2.769	-0.074	-1.918	0.056	-5.608	0.069	
		BMI	-29.376	-0.245	-3.302	0.001	-46.872	-11.880	
Osteopenia (*n=*163)	Model 1	(Intercept)	800.274		11.441	<0.001	662.129	938.418	0.558
		Total Fat mass	0.003	0.084	1.583	0.115	-0.001	0.006	
		Total Muscle mass	0.025	0.730	13.729	<0.001	0.022	0.029	
	Model 2	(Intercept)	1253.251		13.225	<0.001	1066.085	1440.417	0.660
		Total Fat mass	0.014	0.432	5.635	<0.001	0.009	0.019	
		Total Muscle mass	0.029	0.843	16.472	<0.001	0.026	0.033	
		Age	-2.891	-0.142	-3.050	0.003	-4.763	-1.019	
		BMI	-28.155	-0.478	-5.847	<0.001	-37.665	-18.645	
Osteoporosis (*n=*93)	Model 1	(Intercept)	837.300		9.991	<.001	670.801	1003.799	0.339
		Total Fat mass	0.009	0.334	3.844	<.001	0.004	0.013	
		Total Muscle mass	0.012	0.422	4.856	<.001	0.007	0.017	
	Model 2	(Intercept)	1138.461		6.973	<.001	813.993	1462.930	0.382
		Total Fat mass	0.009	0.336	2.985	0.004	0.003	0.015	
		Total Muscle mass	0.011	0.373	4.207	<.001	0.006	0.016	
		Age	-4.415	-0.240	-2.883	0.005	-7.459	-1.371	
		BMI	1.862	0.032	0.273	0.785	-11.674	15.398	

Dependent variable, Bone mineral content.

Model 1 Predictors, (Constant), Total muscle mass, Total fat mass.

Model 2 Predictors, (Constant), Total muscle mass, Total fat mass, Age, BMI.

B, unstandardized regression coefficient; β, standardized regression coefficient; CI, confidence interval; BMI, body mass index.

## Discussion

Based on DXA data from 628 clinical subjects, this study assessed the independent effects of total body muscle mass and fat mass on BMC within a single model, and conducted stratified analyses across different sexes, ages, and bone density statuses. The results indicate a stable and significant positive association between total body muscle mass and BMC, whereas fat mass demonstrated a complex, condition-dependent relationship—showing a certain positive correlation in females, individuals over 70 years old, and the osteoporosis group, but with a weaker or non-significant effect in the total sample and certain subgroups. Furthermore, age was a consistent negative influencing factor for total BMC, with the effect being particularly significant in females, those under 60 years old, and the osteoporosis population; meanwhile, BMI was independently associated with decreased BMC in the overall sample, females, individuals over 70 years old, and in groups with normal or osteopenic bone mass. It is important to note that the “total muscle mass” derived from DXA in this study technically represents lean soft tissue mass, which includes water, connective tissue, and organ mass in addition to contractile skeletal muscle. This methodological constraint should be considered when interpreting the observed associations.

Consistent with previous studies (19, 20), muscle mass was strongly and consistently associated with bone strength. In the total sample and all stratified analyses (by sex, age, and bone density groups) of this study, muscle mass showed a highly significant positive correlation with BMC (all *P* < 0.001), with both the correlation coefficient (r) and regression coefficient (β) being significantly higher than those for fat mass. More importantly, the independent positive association of muscle mass with BMC remained highly significant in both the basic model and extended models adjusted for covariates such as age and BMI. The variability of its standardized regression coefficient (β) was much smaller than that of fat mass, demonstrating a more robust and consistent association of muscle mass with bone. These findings are highly consistent with the theory of muscle-bone coupling, in which muscle contraction stimulates bone through mechanical loading, activating mechanosensing pathways (such as PIEZO ion channels) in bone cells to promote bone formation. Furthermore, various myokines secreted by muscle (such as irisin) and extracellular vesicle-carried miRNAs (e.g., miR-27a-3p) can directly regulate osteogenic differentiation and bone remodeling processes (6, 21). In the population over 70 years old in this study, the association of muscle mass with BMC was the strongest (Model 2 *β=*0.792), suggesting that the association between muscle maintenance and skeletal health may become increasingly prominent with aging. Even in the osteoporosis group, muscle mass remained positively correlated with BMC (*β=*0.373-0.422). Collectively, these observations underscore the importance of muscle mass in bone health and suggest that interventions aimed at maintaining or increasing muscle mass warrant further investigation as a potential strategy for osteoporosis prevention and management.

The role of adipose tissue in skeletal health remains controversial. Ectopic lipid deposition can induce lipotoxicity, promoting osteoclast differentiation from bone marrow progenitors while inhibiting the osteogenic potential of mesenchymal stem cells (MSCs) (22). Concurrently, visceral fat accumulation is often associated with a chronic low-grade inflammatory state (e.g., elevated TNF-α and IL-6), which accelerates bone resorption by enhancing osteoclast activity and suppressing osteoblast function (23). Furthermore, adipokines such as leptin and adiponectin modulate bone metabolism through complex central and peripheral pathways (24–26). As a common indicator of overall obesity (27), BMI demonstrated an independent negative association with BMC in this study. This negative association was observed in the total sample and most subgroups, being particularly pronounced in females, individuals over 70 years of age, and those with osteopenia. This suggests that the metabolic disturbances linked to overall obesity may adversely impact bone health.

Our findings provide a more nuanced perspective on the “obesity paradox”. Although fat mass was correlated with BMC in the total sample and most subgroups, the strength of this association was substantially weaker than that of muscle mass and exhibited clear population heterogeneity. The relationship between fat and bone appears highly dependent on sex, hormonal milieu, and bone metabolic status (28–30). In our study, the association was stronger in females, which may be partly attributed to the role of adipose tissue as a primary source of aromatase for estrogen synthesis. Estrogen promotes osteoblast differentiation and maturation while inhibiting osteoclastogenesis and inducing osteoclast apoptosis (31, 32). Conversely, in adults over 70 years of age and in those with osteoporosis, fat mass emerged as a significant independent positive correlate of BMC. This indicates that during aging or severe bone loss, the metabolic support role of fat and its potential “protective effect” as a mechanical load may become relatively more prominent. Therefore, the net impact of fat on the skeleton represents a complex balance. It is not uniformly beneficial or detrimental but likely depends on the interplay between fat distribution, overall adiposity (BMI), concomitant metabolic and inflammatory status, and individual physiological context. In clinical practice, when assessing body composition in relation to skeletal health, it is essential to move beyond a simplistic focus on total fat mass and instead comprehensively consider the metabolic quality of obesity and the individual’s overall condition.

Age exhibited a significant sex-specific influence on BMC in this study, reflecting the distinct roles of sex hormones in skeletal aging. Among females, age was a strong and independent negative predictor of BMC (β =-0.395, *P* < 0.001), which aligns with accelerated bone turnover and increased bone resorption driven by the sharp decline in estrogen levels after menopause (33). This also explains why women are not only at higher risk for osteoporosis but also experience a much faster rate of bone loss compared to men. In contrast, age was not a significant independent predictor of BMC in males (β =-0.024, *P* = 0.783). This finding is consistent with previous studies indicating that age-related bone loss in men typically begins later, progresses more slowly, and follows a more linear decline (34, 35). This is largely attributed to the absence of a sudden hormonal event analogous to menopause in women; instead, the decline in male sex hormone levels with age is a relatively gradual process (36, 37), which may explain why its association with BMC appears less pronounced in cross-sectional studies. In summary, our data reinforce the central role of estrogen in bone homeostasis and highlight the necessity of tailoring osteoporosis prevention and management strategies to sex-specific aging patterns.

Stratified analysis by bone density status further elucidated the dynamic relationships of muscle and fat with skeletal health. In the normal and osteopenic stages, the regression models showed strong explanatory power (adjusted R²=0.484 and 0.660, respectively). Here, total muscle mass was the strongest and most stable factor positively associated with BMC, whereas the independent association of fat mass was weak or non-significant. This suggests that inadequate mechanical stimulation may be a key driver of bone loss in early disease stages. Consequently, interventions during this phase might focus on enhancing muscle mass and strength—for example, through resistance training—to decelerate mineral loss and improve bone strength (38–40). In the osteoporosis stage, although muscle mass remained a primary correlate of BMC, fat mass also emerged as an independent, significant positive factor, with both contributing jointly to BMC. Notably, the model’s explanatory power declined in this group (adjusted R²=0.382). This shift implies that in established osteoporosis, the dominant link between muscle and bone may attenuate, while adipose tissue might play a relatively greater role, potentially helping to mitigate bone loss through metabolic or endocrine mechanisms. These observations offer a refined perspective for osteoporosis management.

This study highlights the distinct contributions of muscle and fat to skeletal health. Our cross-sectional data reveal a robust, positive association between muscle mass and BMC, reinforcing muscle’s role as a key correlate in bone’s mechanical and metabolic milieu. In contrast, the relationship between fat mass and BMC was highly context-dependent. The positive associations observed in women, older adults, and individuals with osteoporosis suggest that adipose tissue may exert its influence may be related to local hormonal activity (e.g., estrogen synthesis) or energy substrate provision, rather than through a universally protective effect. Thus, in designing strategies to preserve skeletal health, priority should be given to maintaining or increasing muscle mass, whereas the role of fat requires an individualized assessment that moves beyond a simple binary of “beneficial” or “harmful.”

## Limitations

This study has several limitations. First, the cross-sectional design precludes causal inferences regarding the observed associations. Second, the single-center, retrospective design and the pronounced sex imbalance (130 males vs. 498 females) may limit the generalizability of the findings. It is important to note that this sex distribution reflects the real-world epidemiology of osteoporosis in the Chinese population (3), where nationwide data show a significantly higher prevalence in women (32.1% in women over 50 vs. 6.9% in men) leading to a greater proportion of women undergoing DXA screening in clinical practice. Consequently, this imbalance substantially reduces the statistical power for sex-specific analyses, particularly in the male subgroup where the regression models showed low explanatory power (adjusted R²=0.131). Therefore, findings related to sex differences, especially those concerning the role of fat mass in men, should be interpreted with caution and require validation in larger, more balanced cohorts. Third, although statistically significant, the regression models—especially for males (adjusted R²=0.131)-had limited explanatory power, indicating that important confounding or contributing factors (e.g., physical activity, dietary calcium and vitamin D, sex hormone levels, inflammatory markers, and genetic background) were not measured. These unmeasured variables may have influenced the observed associations and could partially explain the limited explanatory power of some subgroup models. Fourth, methodological constraints should be noted: the “total muscle mass” derived from DXA represents lean soft tissue rather than specific skeletal muscle mass or function, and the analysis could not differentiate between adipose tissue depots (e.g., subcutaneous vs. visceral fat), which may have distinct effects on bone metabolism.

Based on these limitations, future studies should: (1) employ longitudinal or interventional designs to establish causality; (2) recruit larger, multi-center cohorts with balanced sex representation to enhance generalizability and statistical power; and (3) integrate detailed measures of lifestyle (diet, exercise), systemic biomarkers (hormones, inflammatory markers), body composition specificity (muscle quality, fat distribution), and omics data to fully elucidate the mechanisms underlying muscle–fat–bone interactions.

## Conclusion

In summary, analyses of DXA body composition data from 628 individuals demonstrate that muscle mass is the strongest and most consistent correlate of BMC, supporting a strong associative relationship between muscle and bone. In contrast, the association of fat mass with BMC is highly context-dependent, appearing most evident in women and patients with osteoporosis, although the sex-specific findings in males require further investigation due to the smaller sample size. Age shows an independent inverse association with BMC in women, underscoring the complexity of postmenopausal bone health. Based on current associative evidence, maintaining muscle mass may be an important consideration in strategies for osteoporosis prevention, while supporting a personalized approach to assessing the role of fat based on individual metabolic and hormonal profiles.

## Data Availability

The original contributions presented in the study are included in the article/**Supplementary Material**. Further inquiries can be directed to the corresponding author/s.
