# Supplementary material for: Assessment of the effects of whole-body muscle and fat mass on bone mineral content based on 628 DXA datasets
Source: Front Endocrinol (Lausanne). 2026 Mar 11;17:1798429. doi: 10.3389/fendo.2026.1798429 (PMC13013020; doi:10.3389/fendo.2026.1798429)
Supplement: Supplementary file 1 [file Table1.docx]

Table 1. Correlation for Total Sample(n=628)

|  |  | Age | Height | Weight | BMI | Total BMC | Total  Fat mass | Total  Muscle mass |
| --- | --- | --- | --- | --- | --- | --- | --- | --- |
| Age | r_ₛ_ | 1 | -0.19 | -0.06 | 0.056 | -0.326 | -0.058 | -0.044 |
|  | *P* | . | <0.001 | 0.158 | 0.160 | <0.001 | 0.146 | 0.269 |
| Height | r_ₛ_ | -0.188 | 1 | 0.5 | -0.013 | 0.640 | 0.041 | 0.655 |
|  | *P* | <0.001 | . | <0.001 | 0.744 | <0.001 | 0.302 | <0.001 |
| Weight | r_ₛ_ | -0.056 | 0.500 | 1 | 0.831 | 0.542 | 0.658 | 0.742 |
|  | *P* | 0.158 | <0.001 | . | <0.001 | <0.001 | <0.001 | <0.001 |
| BMI | r_ₛ_ | 0.056 | -0.013 | 0.831 | 1 | 0.229 | 0.749 | 0.447 |
|  | *P* | 0.16 | 0.744 | <0.001 | . | <0.001 | <0.001 | <0.001 |
| Total BMC | r_ₛ_ | -0.326 | 0.640 | 0.542 | 0.229 | 1 | 0.154 | 0.620 |
|  | *P* | <0.001 | <0.001 | <0.001 | <0.001 | . | <0.001 | <0.001 |
| Total  Fat mass | r_ₛ_ | -0.058 | 0.041 | 0.658 | 0.749 | 0.154 | 1 | 0.155 |
|  | *P* | 0.146 | 0.302 | <0.001 | <0.001 | <0.001 | . | <0.001 |
| Total  Muscle mass | r_ₛ_ | -0.044 | 0.655 | 0.742 | 0.447 | 0.620 | 0.155 | 1 |
|  | *P* | 0.269 | <0.001 | <0.001 | <0.001 | <0.001 | <0.001 | . |

Table 2. Correlation for Females (n=498)

|  |  | Age | Height | Weight | BMI | Total BMC | Total  Fat mass | Total  Muscle mass |
| --- | --- | --- | --- | --- | --- | --- | --- | --- |
| Age | r_ₛ_ | 1 | -0.236 | -0.015 | 0.101 | -0.456 | -0.010 | -0.037 |
|  | *P* | . | <0.001 | 0.740 | 0.025 | <0.001 | 0.828 | 0.409 |
| Height | r_ₛ_ | -0.236 | 1 | 0.340 | -0.094 | 0.456 | 0.164 | 0.454 |
|  | *P* | <0.001 | . | <0.001 | 0.035 | <0.001 | <0.001 | <0.001 |
| Weight | r_ₛ_ | -0.015 | 0.340 | 1 | 0.882 | 0.405 | 0.828 | 0.698 |
|  | *P* | 0.740 | <0.001 | . | <0.001 | <0.001 | <0.001 | <0.001 |
| BMI | r_ₛ_ | 0.101 | -0.094 | 0.882 | 1 | 0.210 | 0.787 | 0.523 |
|  | *P* | 0.025 | 0.035 | <0.001 | . | <0.001 | <0.001 | <0.001 |
| Total BMC | r_ₛ_ | -0.456 | 0.456 | 0.405 | 0.210 | 1 | 0.314 | 0.409 |
|  | *P* | <0.001 | <0.001 | <0.001 | <0.001 | . | <0.001 | <0.001 |
| Total  Fat mass | r_ₛ_ | -0.010 | 0.164 | 0.828 | 0.787 | 0.314 | 1 | 0.353 |
|  | *P* | 0.828 | <0.001 | <0.001 | <0.001 | <0.001 | . | <0.001 |
| Total  Muscle mass | r_ₛ_ | -0.037 | 0.454 | 0.698 | 0.523 | 0.409 | 0.353 | 1 |
|  | *P* | 0.409 | <0.001 | <0.001 | <0.001 | <0.001 | <0.001 | . |

Table 3. Correlation for Males (n=130)

|  |  | Age | Height | Weight | BMI | Total BMC | Total  Fat mass | Total Muscle mass |
| --- | --- | --- | --- | --- | --- | --- | --- | --- |
| Age | r_ₛ_ | 1 | -0.269 | -0.240 | -0.092 | -0.168 | -0.210 | -0.149 |
|  | *P* | . | 0.002 | 0.006 | 0.298 | 0.055 | 0.016 | 0.091 |
| Height | r_ₛ_ | -0.269 | 1 | 0.409 | -0.131 | 0.479 | 0.143 | 0.528 |
|  | *P* | 0.002 | . | <0.001 | 0.138 | <0.001 | 0.106 | <0.001 |
| Weight | r_ₛ_ | -0.240 | 0.409 | 1 | 0.818 | 0.424 | 0.781 | 0.632 |
|  | *P* | 0.006 | <0.001 | . | <0.001 | <0.001 | <0.001 | <0.001 |
| BMI | r_ₛ_ | -0.092 | -0.131 | 0.818 | 1 | 0.168 | 0.760 | 0.378 |
|  | *P* | 0.298 | 0.138 | <0.001 | . | 0.056 | <0.001 | <0.001 |
| Total BMC | r_ₛ_ | -0.168 | 0.479 | 0.424 | 0.168 | 1 | 0.242 | 0.356 |
|  | *P* | 0.055 | <0.001 | <0.001 | 0.056 | . | 0.005 | <0.001 |
| Total  Fat mass | r_ₛ_ | -0.210 | 0.143 | 0.781 | 0.760 | 0.242 | 1 | 0.198 |
|  | *P* | 0.016 | 0.106 | <0.001 | <0.001 | 0.005 | . | 0.024 |
| Total  Muscle mass | r_ₛ_ | -0.149 | 0.528 | 0.632 | 0.378 | 0.356 | 0.198 | 1 |
|  | *P* | 0.091 | <0.001 | <0.001 | <0.001 | <0.001 | 0.024 | . |

Table 4. Correlation for <60 years（n=271）

|  |  | Age | Height | Weight | BMI | Total  BMC | Total  Fat mass | Total  Muscle mass |
| --- | --- | --- | --- | --- | --- | --- | --- | --- |
| Age | r_ₛ_ | 1.000 | -0.186 | -0.111 | -0.008 | -0.320 | -0.085 | -0.089 |
|  | *P* | . | 0.002 | 0.068 | 0.890 | <0.001 | 0.164 | 0.143 |
| Height | r_ₛ_ | -0.186 | 1.000 | 0.506 | 0.008 | 0.661 | 0.044 | 0.614 |
|  | *P* | 0.002 | . | <0.001 | 0.900 | <0.001 | 0.467 | <0.001 |
| Weight | r_ₛ_ | -0.111 | 0.506 | 1.000 | 0.837 | 0.593 | 0.671 | 0.762 |
|  | *P* | 0.068 | <0.001 | . | <0.001 | <0.001 | <0.001 | <0.001 |
| BMI | r_ₛ_ | -0.008 | 0.008 | 0.837 | 1.000 | 0.287 | 0.764 | 0.486 |
|  | *P* | 0.890 | 0.900 | <0.001 | . | <0.001 | <0.001 | <0.001 |
| Total  BMC | r_ₛ_ | -0.320 | 0.661 | 0.593 | 0.287 | 1.000 | 0.161 | 0.607 |
|  | *P* | <0.001 | <0.001 | <0.001 | <0.001 | . | 0.008 | <0.001 |
| Total  Fat mass | r_ₛ_ | -0.085 | 0.044 | 0.671 | 0.764 | 0.161 | 1.000 | 0.168 |
|  | *P* | 0.164 | 0.467 | <0.001 | <0.001 | 0.008 | . | 0.006 |
| Total  Muscle mass | r_ₛ_ | -0.089 | 0.614 | 0.762 | 0.486 | 0.607 | 0.168 | 1.000 |
|  | *P* | 0.143 | <0.001 | <0.001 | <0.001 | <0.001 | 0.006 | . |

Table 5. Correlation for 60-70 years（n=229）

|  |  | Age | Height | Weight | BMI | Total  BMC | Total  Fat mass | Total  Muscle mass |
| --- | --- | --- | --- | --- | --- | --- | --- | --- |
| Age | r_ₛ_ | 1.000 | -0.077 | 0.065 | 0.106 | -0.063 | 0.103 | 0.008 |
|  | *P* | . | 0.248 | 0.326 | 0.110 | 0.343 | 0.121 | 0.901 |
| Height | r_ₛ_ | -0.077 | 1.000 | 0.454 | -0.050 | 0.574 | -0.020 | 0.683 |
|  | *P* | 0.248 | . | <0.001 | 0.449 | <0.001 | 0.769 | <0.001 |
| Weight | r_ₛ_ | 0.065 | 0.454 | 1.000 | 0.836 | 0.514 | 0.662 | 0.709 |
|  | *P* | 0.326 | <0.001 | . | <0.001 | <0.001 | <0.001 | <0.001 |
| BMI | r_ₛ_ | 0.106 | -0.050 | 0.836 | 1.000 | 0.241 | 0.770 | 0.404 |
|  | *P* | 0.110 | 0.449 | <0.001 | . | <0.001 | <0.001 | <0.001 |
| Total  BMC | r_ₛ_ | -0.063 | 0.574 | 0.514 | 0.241 | 1.000 | 0.141 | 0.642 |
|  | *P* | 0.343 | <0.001 | <0.001 | <0.001 | . | 0.033 | <0.001 |
| Total  Fat mass | r_ₛ_ | 0.103 | -0.020 | 0.662 | 0.770 | 0.141 | 1.000 | 0.144 |
|  | *P* | 0.121 | 0.769 | <0.001 | <0.001 | 0.033 | . | 0.029 |
| Total  Muscle mass | r_ₛ_ | 0.008 | 0.683 | 0.709 | 0.404 | 0.642 | 0.144 | 1.000 |
|  | *P* | 0.901 | <0.001 | <0.001 | <0.001 | <0.001 | 0.029 | . |

Table 6. Correlation for >70 years（n=128）

|  |  | Age | Height | Weight | BMI | Total  BMC | Total  Fat mass | Total  Muscle mass |
| --- | --- | --- | --- | --- | --- | --- | --- | --- |
| Age | r_ₛ_ | 1.000 | -0.136 | -0.033 | 0.033 | -0.098 | -0.025 | -0.072 |
|  | *P* | . | 0.125 | 0.713 | 0.713 | 0.273 | 0.777 | 0.417 |
| Height | r_ₛ_ | -0.136 | 1.000 | 0.539 | 0.048 | 0.629 | 0.092 | 0.694 |
|  | *P* | 0.125 | . | <0.001 | 0.590 | <0.001 | 0.300 | <0.001 |
| Weight | r_ₛ_ | -0.033 | 0.539 | 1.000 | 0.839 | 0.569 | 0.621 | 0.749 |
|  | *P* | 0.713 | <0.001 | . | <0.001 | <0.001 | <0.001 | <0.001 |
| BMI | r_ₛ_ | 0.033 | 0.048 | 0.839 | 1.000 | 0.274 | 0.709 | 0.450 |
|  | *P* | 0.713 | 0.590 | <0.001 | . | 0.002 | <0.001 | <0.001 |
| Total  BMC | r_ₛ_ | -0.098 | 0.629 | 0.569 | 0.274 | 1.000 | 0.167 | 0.701 |
|  | *P* | 0.273 | <0.001 | <0.001 | 0.002 | . | 0.059 | <0.001 |
| Total  Fat mass | r_ₛ_ | -0.025 | 0.092 | 0.621 | 0.709 | 0.167 | 1.000 | 0.159 |
|  | *P* | 0.777 | 0.300 | <0.001 | <0.001 | 0.059 | . | 0.074 |
| Total  Muscle mass | r_ₛ_ | -0.072 | 0.694 | 0.749 | 0.450 | 0.701 | 0.159 | 1.000 |
|  | *P* | 0.417 | <0.001 | <0.001 | <0.001 | <0.001 | 0.074 | . |

Table 7. Correlation for Normal Bone Mass (n=372)

|  |  | Age | Height | Weight | BMI | Total  BMC | Total  Fat mass | Total  Muscle mass |
| --- | --- | --- | --- | --- | --- | --- | --- | --- |
| Age | r_ₛ_ | 1 | -0.102 | 0.016 | 0.087 | -0.096 | -0.083 | 0.085 |
|  | *P* | . | 0.049 | 0.757 | 0.095 | 0.065 | 0.109 | 0.103 |
| Height | r_ₛ_ | -0.102 | 1 | 0.498 | -0.010 | 0.689 | -0.030 | 0.669 |
|  | *P* | 0.049 | . | <0.001 | 0.843 | <0.001 | 0.558 | <0.001 |
| Weight | r_ₛ_ | 0.016 | 0.498 | 1 | 0.835 | 0.611 | 0.622 | 0.749 |
|  | *P* | 0.757 | <0.001 | . | <0.001 | <0.001 | <0.001 | <0.001 |
| BMI | r_ₛ_ | 0.087 | -0.01 | 0.835 | 1 | 0.279 | 0.751 | 0.446 |
|  | *P* | 0.095 | 0.843 | <0.001 | . | <0.001 | <0.001 | <0.001 |
| Total  BMC | r_ₛ_ | -0.096 | 0.689 | 0.611 | 0.279 | 1 | 0.126 | 0.690 |
|  | *P* | 0.065 | <0.001 | <0.001 | <0.001 | . | 0.015 | <0.001 |
| Total  Fat mass | r_ₛ_ | -0.083 | -0.030 | 0.622 | 0.751 | 0.126 | 1 | 0.089 |
|  | *P* | 0.109 | 0.558 | <0.001 | <0.001 | 0.015 | . | 0.087 |
| Total  Muscle mass | r_ₛ_ | 0.085 | 0.669 | 0.749 | 0.446 | 0.690 | 0.089 | 1 |
|  | *P* | 0.103 | <0.001 | <0.001 | <0.001 | <0.001 | 0.087 | . |

Table 8. Correlation for Osteopenia (n=163)

|  |  | Age | Height | Weight | BMI | Total  BMC | Total  Fat mass | Total  Muscle mass |
| --- | --- | --- | --- | --- | --- | --- | --- | --- |
| Age | r_ₛ_ | 1 | -0.108 | 0.009 | 0.106 | -0.180 | -0.012 | -0.018 |
|  | *P* | . | 0.170 | 0.908 | 0.180 | 0.022 | 0.882 | 0.820 |
| Height | r_ₛ_ | -0.108 | 1 | 0.386 | -0.165 | 0.683 | 0.007 | 0.631 |
|  | *P* | 0.170 | . | <0.001 | 0.035 | <0.001 | 0.927 | <0.001 |
| Weight | r_ₛ_ | 0.009 | 0.386 | 1 | 0.814 | 0.572 | 0.728 | 0.714 |
|  | *P* | 0.908 | <0.001 | . | <0.001 | <0.001 | <0.001 | <0.001 |
| BMI | r_ₛ_ | 0.106 | -0.165 | 0.814 | 1 | 0.200 | 0.774 | 0.389 |
|  | *P* | 0.180 | 0.035 | <0.001 | . | 0.011 | <0.001 | <0.001 |
| Total  BMC | r_ₛ_ | -0.180 | 0.683 | 0.572 | 0.200 | 1 | 0.264 | 0.686 |
|  | *P* | 0.022 | <0.001 | <0.001 | 0.011 | . | <0.001 | <0.001 |
| Total  Fat mass | r_ₛ_ | -0.012 | 0.007 | 0.728 | 0.774 | 0.264 | 1 | 0.214 |
|  | *P* | 0.882 | 0.927 | <0.001 | <0.001 | <0.001 | . | 0.006 |
| Total  Muscle mass | r_ₛ_ | -0.018 | 0.631 | 0.714 | 0.389 | 0.686 | 0.214 | 1 |
|  | *P* | 0.820 | <0.001 | <0.001 | <0.001 | <0.001 | 0.006 | . |

Table 9. Correlation for Osteoporosis (n=93)

|  |  | Age | Height | Weight | BMI | Total BMC | Total Fat mass | Total Muscle mass |
| --- | --- | --- | --- | --- | --- | --- | --- | --- |
| Age | r_ₛ_ | 1 | -0.078 | 0.018 | 0.054 | -0.26 | 0.101 | -0.055 |
|  | *P* | . | 0.457 | 0.862 | 0.610 | 0.012 | 0.337 | 0.599 |
| Height | r_ₛ_ | -0.078 | 1 | 0.437 | 0.004 | 0.579 | 0.243 | 0.444 |
|  | *P* | 0.457 | . | <0.001 | 0.971 | <0.001 | 0.019 | <0.001 |
| Weight | r_ₛ_ | 0.018 | 0.437 | 1 | 0.877 | 0.577 | 0.709 | 0.612 |
|  | *P* | 0.862 | <0.001 | . | <0.001 | <0.001 | <0.001 | <0.001 |
| BMI | r_ₛ_ | 0.054 | 0.004 | 0.877 | 1 | 0.336 | 0.646 | 0.477 |
|  | *P* | 0.610 | 0.971 | <0.001 | . | <0.001 | <0.001 | <0.001 |
| Total BMC | r_ₛ_ | -0.26 | 0.579 | 0.577 | 0.336 | 1 | 0.378 | 0.601 |
|  | *P* | 0.012 | <0.001 | <0.001 | <0.001 | . | <0.001 | <0.001 |
| Total Fat mass | r_ₛ_ | 0.101 | 0.243 | 0.709 | 0.646 | 0.378 | 1 | 0.199 |
|  | *P* | 0.337 | 0.019 | <0.001 | <0.001 | <0.001 | . | 0.056 |
| Total Muscle mass | r_ₛ_ | -0.055 | 0.444 | 0.612 | 0.477 | 0.601 | 0.199 | 1 |
|  | *P* | 0.599 | <0.001 | <0.001 | <0.001 | <0.001 | 0.056 | . |
